# Correction: Facile one-pot synthesis of silver nanoparticles encapsulated in natural polymeric urushiol for marine antifouling

**DOI:** 10.1039/d0ra90068k

**Published:** 2020-06-25

**Authors:** Lu Zheng, Yucai Lin, Donghui Wang, Jipeng Chen, Ke Yang, Binbin Zheng, Weibin Bai, Rongkun Jian, Yanlian Xu

**Affiliations:** College of Chemistry and Materials, Fujian Normal University Fuzhou 350007 P. R. China ylxu@fjnu.edu.cn +86 59183464353; Fujian Provincial Key Laboratory of Polymer Materials Fuzhou 350007 P. R. China; Fujian Provincial Key Laboratory of Advanced Oriented Chemical Engineering Fuzhou 350007 P. R. China; Fujian Engineering Research Center of New Chinese Lacquer Material, Minjiang University Fuzhou 350007 P. R. China

## Abstract

Correction for ‘Facile one-pot synthesis of silver nanoparticles encapsulated in natural polymeric urushiol for marine antifouling’ by Lu Zheng *et al.*, *RSC Adv.*, 2020, **10**, 13936–13943, DOI: 10.1039/D0RA02205E.

The authors regret that an incorrect version of Fig. 2 was included in the original article. The correct version of Fig. 2 is presented below.

**Fig. 2:**
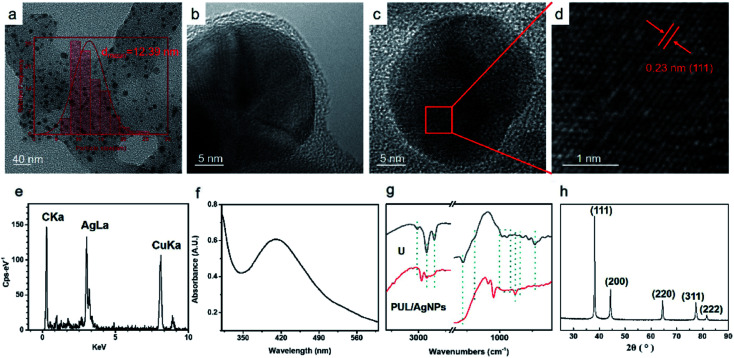
TEM images of PUL/AgNPs (a–d); EDS of AgNPs (e); UV-vis spectra of AgNPs (f); FT-IR spectra of urushiol (U); PUL/AgNPs (g); XRD image of AgNPs (h).

The Royal Society of Chemistry apologises for these errors and any consequent inconvenience to authors and readers.

## Supplementary Material

